# Banking of AT-MSC and its Influence on Their Application to Clinical Procedures

**DOI:** 10.3389/fbioe.2021.773123

**Published:** 2021-11-30

**Authors:** Ekaterina Semenova, Mariusz P. Grudniak, Katarzyna Bocian, Magdalena Chroscinska-Krawczyk, Marzena Trochonowicz, Igor M. Stepaniec, Magdalena Murzyn, Ilona Szablowska-Gadomska, Dariusz Boruczkowski, Tomasz Oldak, Eugeniusz K. Machaj

**Affiliations:** ^1^ Polish Stem Cell Bank, FamiCord Group, Warsaw, Poland; ^2^ Department of Immunology, Faculty of Biology, University of Warsaw, Warsaw, Poland; ^3^ Clinic of Paediatric Neurology, III Faculty of Paediatrics, Medical University of Lublin, Lublin, Poland; ^4^ Institute of Human Genetics, Polish Academy of Sciences, Poznan, Poland; ^5^ Laboratory for Cell Research and Application, Medical University of Warsaw, Warsaw, Poland

**Keywords:** cryopreservation, banking, cryoprotectant, ADSC, application

## Abstract

Processing of MSCs to obtain a therapeutic product consists of two main steps: 1) the *in vitro* expansion of the cells until an appropriate number of them is obtained, and 2) freezing and storage of the expanded cells. The last step is critical and must be optimized so that after thawing the cells retain all their physiological properties including the secretory function. In this paper, we evaluated physiological parameters of AT-MSC’s after a full cycle of their processing, particularly freezing and storing at the liquid nitrogen vapor temperature. Based on the recovered proliferative and secretory capacities of the thawed cells, we have designed the optimal technique for processing of MSCs for clinical applications. In our work, we tried to select the best DMSO-based cryoprotectant mixture on the base of post thawing fully retain their properties. We have demonstrated the effectiveness of the use of DMSO in various configurations of the constituent cryoprotective fluids. We have also shown that AT-MSCs that show control levels in most standard tests (viability, shape, culture behaviour, and proliferative properties) after thawing, may show transient variations in some important physiological properties, such as the level of secreted growth factors. Obtained results let us to indicate how to optimize the AT-MSC preparation process for clinical applications. We suggest that before their clinical application the cells should be cultured for at least one passage to recover their physiological stability and thus assure their optimal therapeutic potential.

## Introduction

Adipose tissue-derived mesenchymal stromal cells (AT-MSCs) and MSCs from other sources possess such attributes as adherence to surfaces, no surface expression of the CD14, CD19, CD34, CD45, HLA-DR antigens, expression of the CD73, CD90, CD105 antigens and the capacity to undergo adipogenic, osteogenic and chondrogenic differentiation (Dominici et al., 2006). MSCs secrete various growth factors, such as tumor necrosis factor-alpha (TNF-α), transforming growth factor-beta (TGF-β), vascular endothelial growth factor (VEGF) and hepatocyte growth factor (HGF), chemokines MCP-1 and IL-8, and cytokines IL-3, IL-5 and IL-6 (Salgado et al., 2010; Carmeliet and Jain, 2011; Doorn et al., 2012; Reinders et al., 2013; Schepers and Fibbe, 2016). MSCs cells also tend to migrate to the sites of injury or inflammation (Spaeth et al., 2008; Rustad and Gurtner, 2012; Ullah et al., 2019). Due to these unique properties AT-MSCs are widely used in regenerative medicine and other clinical applications (Wang et al., 2012; Kim and Cho, 2013; Semenova et al., 2018; Han et al., 2019; Pittenger et al., 2019). The therapeutic mechanism of these cells has not been fully elucidated, but is likely associated with either direct interaction between MSCs and the recipient cells or stimulation of the recipient tissue by soluble factors secreted by MSCs. In the former case, the main role is played by the MSCs’ capacity to differentiate into many different types of cells. In the latter case, the secretory function of MSCs is likely to play a major role.

Processing of MSCs to obtain a therapeutic product consists of two main steps: 1) the *in vitro* expansion of the cells until an appropriate number of them is obtained, and 2) freezing and storage of the expanded cells. The last step is critical and must be optimized so that after thawing the cells retain all their physiological properties including the secretory function. Of crucial importance is maintenance of the freezing rate at about 1°C per minute as is the appropriate selection of a cryoprotective solution. The latter should effectively prevent formation of ice crystals inside and outside of the cells, keep them viable during the freezing and storing and save them physiological properties intact after thawing. For all these purposes dimethyl sulfoxide (DMSO) seems to be the agent of choice (Davis et al., 1990; Stroncek et al., 1991; Lane and Gardner, 2005; Benkhalifa et al., 2014; Cantero Peral et al., 2015; Truong et al., 2016; Kang et al., 2017).

In this paper, we evaluated physiological parameters of AT-MSC’s after a full cycle of their processing, particularly freezing and storing at the liquid nitrogen vapor temperature. Based on the recovered proliferative and secretory capacities of the thawed cells, we have designed the optimal technique for processing of MSCs for clinical applications.

## Materials and Methods

### Cell Isolation and Culture

Adipose tissue samples were collected in accordance with a protocol approved by the Polish Ministry of Health and proceeded within 24 h after the collection. The samples were obtained anonymously from healthy volunteers (*n* = 5) by liposuction. Every sample of adipose tissue (lipoaspirate) was 100–150 ml of capacity. The lipoaspirate was washed several times until a clear yellow tissue was obtained. The tissue was then digested with 0.1% collagenase NB 6 (SERVA) for about 30–60 min at 37°C with a rotation (depending on the quality of the lipoaspirate and content of adipose tissue in it, the incubation time varies. The criterion for interrupting the digestion process is the achievement of the appropriate consistency and homogeneity by the processed lipoaspirate). The obtained suspension was centrifuged (300 g, 7 min) to remove the collagenase and to isolate the stromal vascular fraction (SVF), the heterogeneous cell population consisting with preadipocytes, macrophages, lymphocytes, endothelial cells, AT-MSC and many others. SVF was then suspended in StemMACS™ human MSC Expansion Media Kit XF (Miltenyi Biotec) supplemented with antibiotics/antimicotics (Thermo Fisher) and adhered AT-MSCs were expanded in the 75 cm^2^ flasks (NUNC, Thermo Scientific) at 37°C, 5% CO_2_ and 90% humidity. AT-MSCs were cultured in these standard conditions until passage 3, which was used in further experiments.

### Freezing and Thawing Procedures

The freezing procedure was performed using the IceCube device (Sylab). Four DMSO-based cryoprotectants were selected for our purposes: **Cryo1**—commercially available from Biological Industries; **Cryo2**—commercially available from ZENOAQ/AMSBIO; **Cry3**—composed of 10% DMSO, dextran40 in NaCl and 5% human albumin Alburex 5 (CSL Bechring) (Gao and Critser, 2000; Fuller, 2004); **Cryo4**—composed of 10% DMSO in Alburex 5.

**FIGURE 1:**
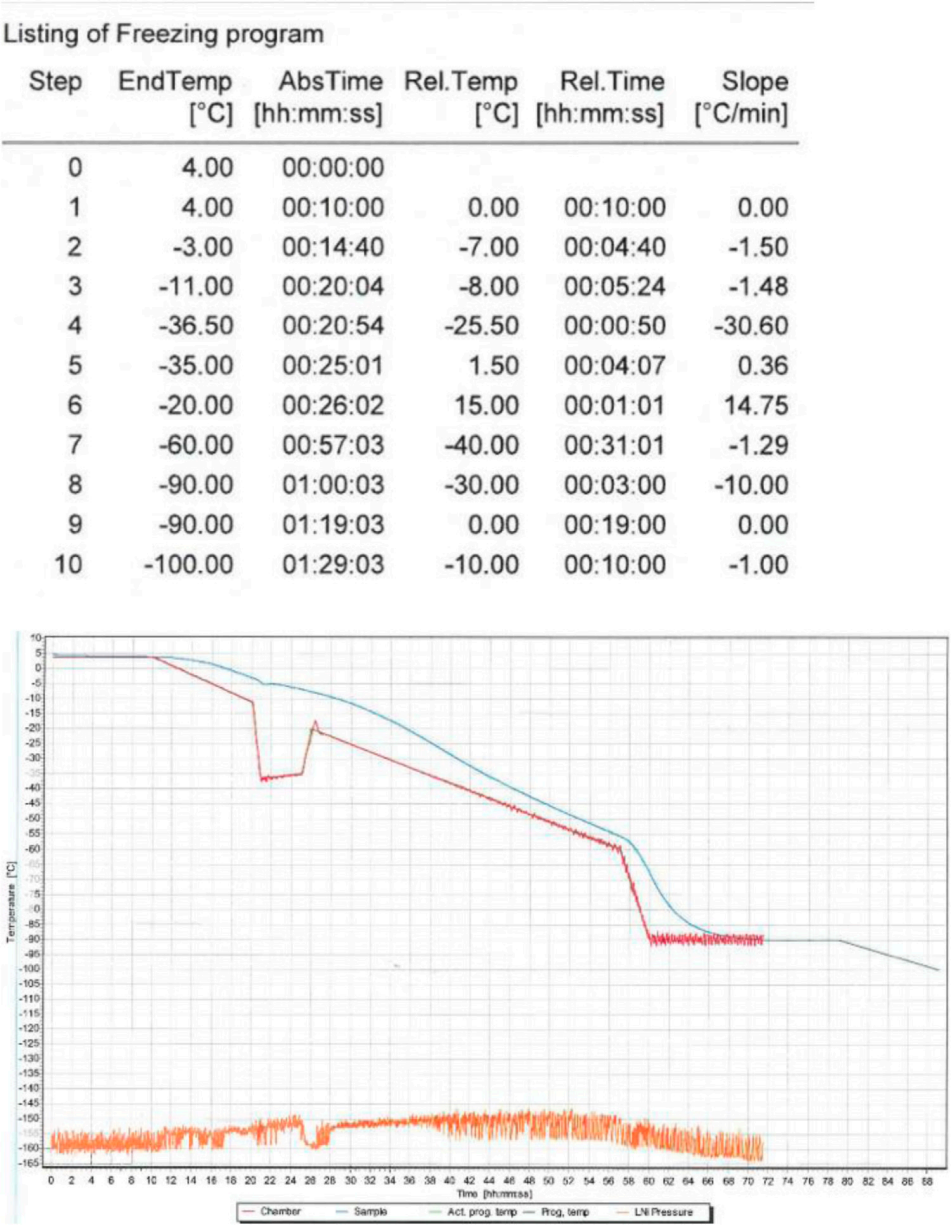
The freezing procedure was implemented using the IceCube device. The freezing program started at 4°C and finished at −80°C. After freezing AT-MSCs were transferred to cryogenic containers (−170°C).

For the experiments AT-MSCs from the third passage (P3) were used. The cells were frozen at the density of 5 × 10⁶ cell/ml using our own freezing program formulated for the 2–4.5 ml tubes (Figure 1).

After an arbitrarily selected weekly cryopreservation period, all samples were placed in the 37°C water bath for rapid thawing until no ice was detectable. The thawed samples were slowly dissolved in the culture medium (1:20) and after centrifugation the supernatant containing DMSO was removed. The obtained cells were suspended in the medium using the same seeding density and cultured at 37°C, 5% CO2 and 90% humidity in the 75 cm^2^ flasks (NUNC, Thermo Scientific) until the 5th passage. Non-frozen AT-MSCs from passages 4 and 5 were used as control.

### Flow Cytometry Analysis

The phenotypic profile of AT-MSCs was determined using the PE-conjugated antibodies against human antigens CD73 (catalog no. 550257, BD Pharmingen™, BD Bioscence United States), CD90 (catalog no. 561970, BD Pharmingen™, BD Bioscence United States) and CD105 (catalog no. 560839, BD Pharmingen™, BD Bioscence United States) and using the FITC-conjugated antibodies against human antigens (later referred as Lin cocktail) HLA-DR (catalog no. 555811, BD Pharmingen™, BD Bioscence United States), CD34 (catalog no. 345801, BD Pharmingen™, BD Bioscence United States), CD45 (catalog no. 345808, BD Pharmingen™, BD Bioscence United States), CD 19 (catalog no. 345788, BD Pharmingen™, BD Bioscence United States) and CD14 (catalog no. 345784, BD Pharmingen™, BD Bioscence United States) (BD Bioscience) according to protocol. At least 10 × 10^3^ cells per sample were analyzed in a FACSCalibur cytometer (BD Bioscience). The results were analysed with use of the CellQuest Pro v.6.0 software (BD Bioscence).

### Proliferative Potential—The PDT and CFU-F Assays

The proliferative potential of the cells was evaluated using the population doubling time (PDT) test. The results were calculated using the following formula: PDT = (CT*Ln2)/ln (Nf/Ni), where CT is the cell-culture time, Nf—the final number of cells, and Ni—the initial number of cells.

The colony-forming unit-fibroblast (CFU-F) assay was used for estimation of the cells’ proliferation capacity. The cells from each sample suspended in MACS medium were placed in the 12-well culture plates (NUNC, Thermo Scientific) at 500 cells/well. On day 5 of the culture the cells were fixed with 4% PFA (CHEMLAND) and stained with crystal violet (Gibco). Colonies containing at least 50 cells were counted.

### Post-Thaw Cell Viability

The frozen cells were thawed in the 37°C water bath, centrifuged (300 g, 7 min) to remove the remaining cryoprotectant and suspended in the MACS medium. After 30 min of incubation at 37°C to restore their membranes the cells’ viability was estimated using the Trypan Blue test and the Nikon Eclipse E 200 (Nikon) light microscope at ×20 magnification. Using hemocytometer chamber all living and dead cells are counted and the alive are presented as a percentage of all cells.

### Cytoskeleton Analysis

Cell suspensions obtained from both fresh and frozen/thawed P4 cells were seeded in the 6-well culture plates (NUNC, Thermo Scientific) at 1 × 10^4^ cells per well and cultured until about 60% confluency. The coverslips were removed and the attached cells were fixed with 4% PFA. The cells were then permeabilized with Triton X-100 (Merck) and blocked with 1% BSA in the phosphate-buffered saline (PBS) for 30 min. The obtained cell suspensions were incubated with the primary antibody tagged with the TRITC-conjugated Phalloidin (Merck) for 60 min at room temperature, washed with a washing buffer (1xPBS containing 0.05% Tween-20) and incubated with the FITC-conjugated secondary antibody for 60 min. To stain the nuclei, the cells were additionally incubated with DAPI for 5 min at room temperature. The cell preparations were then examined under the Leica DMi8 (Leica) microscope.

### Multilineage Differentiation and Staining

The P4 cells were seeded at 5 × 10³ cells/cm^2^ onto the wells of the 24-well plate (BD Falcon), grown to the 50% confluence and then cultured in the following media (Thermo Fisher): 1) the StemPro Adipogenesis Differentiation Medium for the evaluation of the cells’ adipogenic potential, 2) the StemPro Chondrogenesis Differentiation Medium for the evaluation of the chondrogenic potential, and 3) the StemPro Osteogenesis Differentiation Medium for the evaluation of the osteogenic potential. After 3 weeks of incubation in standard conditions the cultured cells were fixed with 4% Paraformaldehyde, stained with suitable dye and analyzed under the Leica DM IL LED light microscope (Leica) for the presence of adipocytes (after staining with Oil Red O, Sigma), chondrocytes (after staining with Alcain Blue, Sigma) and osteocytes (after staining with Alizarin Red, Sigma).

### Gene Expression Analysis

The expression of genes was analyzed with qPCR performed in triplicates from three independent experiments. Total RNA was isolated using the RNeasy mini kit (Qiagen, Valencia, CA) from the control and frozen/thawed AT-MSCs (P4 and P5 cells). cDNA was synthesized using the Maxima H Minus First Strand cDNA Synthesis Kit (Thermo Scientific, United States) according to the manufacturer’s instruction. After the reverse transcription, all the cDNA samples were diluted to a final concentration of 8.333 ng/μl and stored at −80°C until further use. The specified primers are presented in Table 1. PowerUp™ SYBR™ Green Master Mix (Thermo Fischer) was used under the following conditions: activation—95°C for 2 min, denaturation—95°C for 5 s, annealing—60°C for 30 s for a total of 40 cycles. Results were analyzed using the 2^−∆Ct^ method relating gene expression to glyceraldehyde-3-phosphate dehydrogenase (GAPDH) and hypoxanthine phosphoribosyltransferase 1 (HPRT 1).

**TABLE 1:** Primers used in the qPCR.

Target gen	Primer	Sequence (5′--> 3′)
ADIPO		
ADIPOQ	F	GCA​GTC​TGT​GGT​TCT​GAT​TCC
	R	CCC​TTG​AGT​CGT​GGT​TTC​CT
FABP4	F	GAA​AGG​CGT​CAC​TTC​CAC​GA
	R	ATG​CGA​ACT​TCA​GTC​CAG​GTC
CHONDRO		
COL10A1	F	GCT​GAA​CGA​TAC​CAA​ATG​CCC
	R	CCT​TGC​TCT​CCT​CTT​ACT​GCT
COL2A1	F	TGGTGCTGCTGACGCT
	R	CTG​TCC​CTT​TGG​TCC​TGG​TT
OSTEO		
RUNX2	F	ACGGGGCACTGGGCTT
	R	GTG​AGG​GAT​GAA​ATG​CTT​GGG
BGLAP	F	CCT​CAC​ACT​CCT​CGC​CCT​AT
	R	CTC​TTC​ACT​ACC​TCG​CTG​CC
PLURI		
SOX-2	F	CGG​AAA​ACC​AAG​ACG​CTC​A
	R	GACCCCGCTCGCCAT
C-MYC	F	CGCCTTCTCTCCGTCCTC
	R	TCT​TCC​TCA​TCT​TCT​TGT​TCC​TCC

Note: ADIPO—genes associated with adipogenesis, CHONDRO—genes associated with chondrogenesis, OSTEO—genes associated with osteogenesis, PLURI—genes associated with pluripotency.

### Multiplex

Supernatants from the P4 and P5 cells were collected, centrifuged (10,000 g, 10 min) and stored at −80°C. According to the producer’s guideline, the MILLIPLEX MAP HCYTOMAG-60K and the TGF-β1,2,3 magnetic bead kits (Merck KGaA) were used to evaluate the following cytokines, chemokines, and growth factors: the pro-inflammatory IL-8, IFN-γ, IL-2, IL-1β, MCP-1, IL-3, IL-12, IL-17, IP-10, MIP-1β, and MIP-1α, the anti-inflammatory IL-4, IL-5, IL-6, IL-10, IL-13, IFN-α, TGF-β1, and TGF-β2, and other growth factors, such as PDGF AA, and G-CSF. All the reagents and samples were prepared according to the manufacturer’s protocol. The Belysa 1.0.19 program was used for the analysis of the results.

### Statistical Analysis

Results are presented as means ± standard error of the mean (SEM). The GraphPad Prism V6.0 software (GraphPad Software, La Jolla, CA) was used to perform *t*-tests and estimate statistical differences between the results. *p-*values less than 0.05 were considered statistically significant.

## Results

### Morphology and Phenotype

**FIGURE 2:**
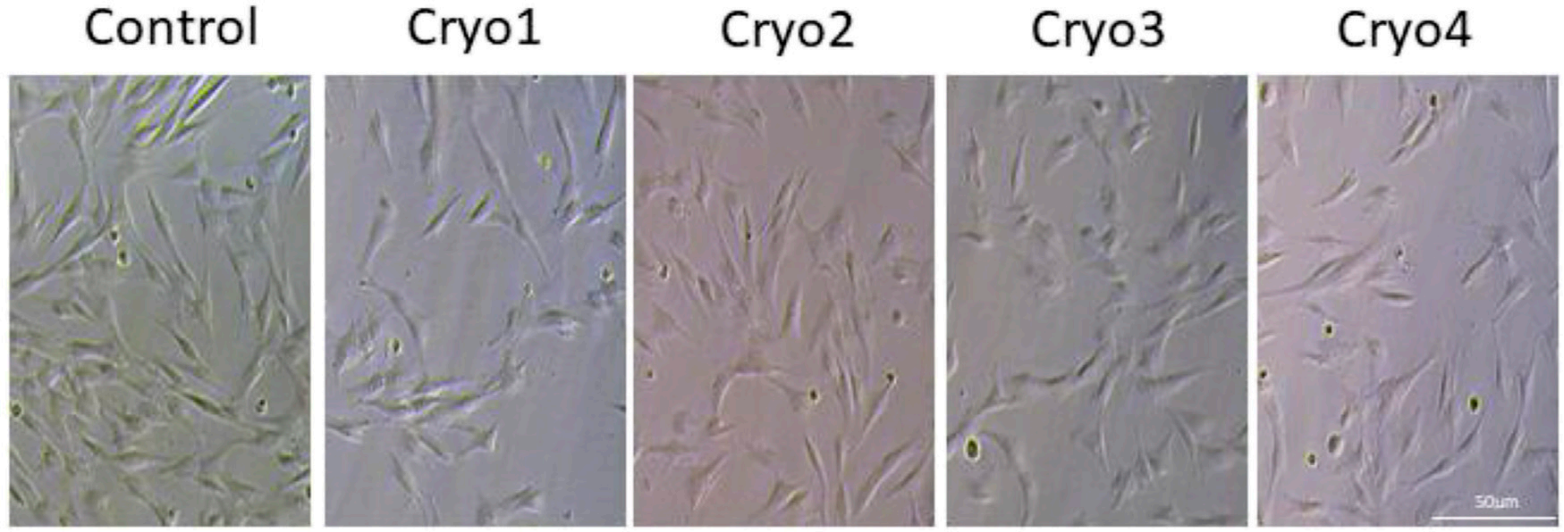
Morphology of cultured MSCs without freezing (Control) and after cryopreservation with four cryoprotectants. The cells were grown attached to the bottom of the flasks and exhibited typical morphology of mesenchymal cells.

Cells from all the groups exhibited a typical fibroblast-like, spindle-shaped morphology (Figure 2) and adhered to plastic surfaces. No marked differences between the non-frozen (control) and frozen/thawed cells were noted with respect to these features.

Cells from the four experimental groups as well as from the control group demonstrated classical phenotype of cultured AT-MSCs, i.e., expressed on the surface the CD73, CD90, CD105 antigens and did not express the CD14, CD19, CD34, CD45, HLA-DR antigens (Lin cocktail) (Table 2). No significant differences in the expression of these antigens were detected between the groups.

**TABLE 2:** Flow cytometry of non-frozen (Control) and frozen/thawed (Cryo1 through Cryo4) AT-MSCs. The Lin cocktail contained antibodies against the CD34, CD45, CD19, CD14, HLA-DR antigens. The flow cytometry analysis of the surface antigen profile was performed from 5 samples.

Control	Lin cocktail	CD73	CD90	CD105
P3	0.35 ± 0.04	98.38 ± 4.1	99.85 ± 13.1	96.73 ± 9.03
P4	0.72 ± 0.02	98.18 ± 10.1	99.67 ± 10.5	96.38 ± 8.1
P5	1.84 ± 0.4	96.2 ± 11.7	95.18 ± 6.8	98.43 ± 6.1
Cryo1	Lin cocktail	CD73	CD90	CD105
After thawing	0.9 ± 1.8	96.67 ± 12.5	99.08 ± 7.23	77.14 ± 12.1
P4	0.13 ± 0.08	99.32 ± 8.1	98.92 ± 9.1	94.93 ± 8.1
P5	0.1 ± 0.03	93.31 ± 9.6	95.84 ± 11.1	92.06 ± 5.6
Cryo2	Lin cocktail	CD73	CD90	CD105
After thawing	0.2 ± 1.22	98.02 ± 10.6	99.08 ± 6.3	84.29 ± 12.3
P4	0.1 ± 0.03	99.02 ± 15.1	99.1 ± 16.2	96.37 ± 7.15
P5	0.01	90.73 ± 7.3	99.08 ± 10.6	93.11 ± 8.27
Cryo3	Lin cocktail	CD73	CD90	CD105
After thawing	0.3 ± 1	96.6 ± 13.5	99.01 ± 10.9	72.64 ± 5.2
P4	0.03	99.6 ± 9.5	99.72 ± 21.6	88.08 ± 9.2
P5	0	88.17 ± 12.3	98.91 ± 9.7	93.29 ± 4.1
Cryo4	Lin cocktail	CD73	CD90	CD105
After thawing	0.7 ± 0.92	98.86 ± 10.6	98.13 ± 12.2	90.95 ± 11.1
P4	0.6 ± 0.01	99.47 ± 15.5	85.72 ± 9.4	85.92 ± 9.3
P5	0	93.46 ± 11.7	99.09 ± 12	86.87 ± 8.8

### Proliferative Potential: PDT and CFU-F Assays

**FIGURE 3:**
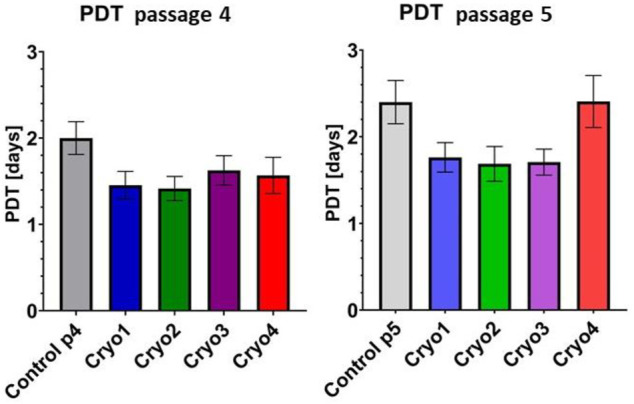
Comparison of PDTs of the non-frozen (Control) and frozen with cryoprotectans 1 through 4 (Cryo 1 through Cryo 4) AT- MSCs from passages 4 and 5. Mean values ± SEM are shown. The PDT analysis was performed from 5 samples.

Proliferative capacities of the control and frozen/thawed AT-MSCs are presented in Figure 3. In the cryopreserved cells obtained from passage 4 the population doubling times were slightly (but not significantly) shorter than that of the control cells (Figure 3, left side). Likewise, PDTs of the cells from passage 5, which were treated with cryoprotectants 1 through 3 were insignificantly less than in the control cells and comparable to those of the P4 cells. In contrast, in the passage 5 cells pre-treated with cryoprotectant 4 PDT was longer than in cells treated with cryoprotectans 1 through 3, but the differences were not statistically significant (Figure 3, right side).

**FIGURE 4:**
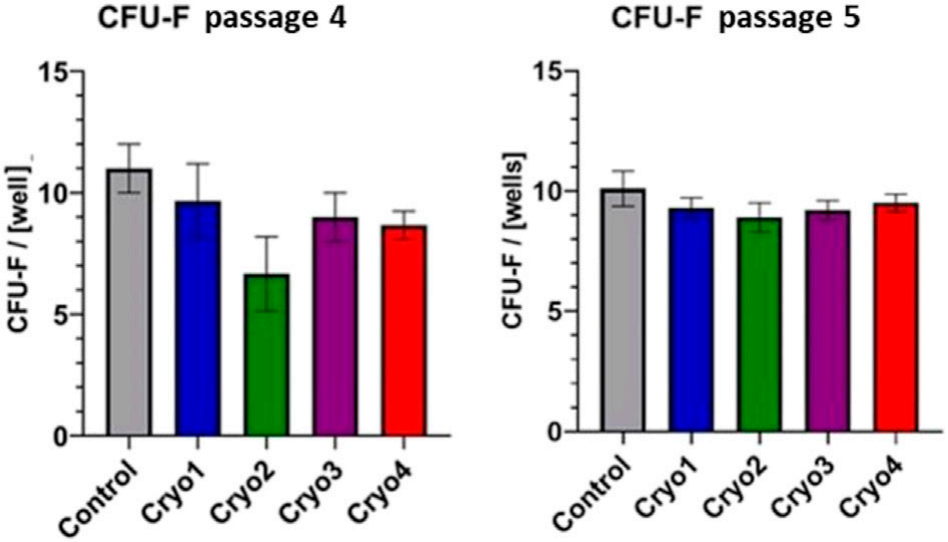
Clonogenic potential of the non-frozen (Control) and frozen/thawed (Cryo 1 through Cryo 4) AT-MSCs obtained from passages 4 and 5. Mean values ± SEM are shown. The CFU-F analysis was performed from 5 samples.

As indicated in Figure 4 clonogenic potential of the non-frozen (Control) cells was slightly higher than those of the frozen/thawed cells. In the passage 4 cells clonogenic potential of cells treated with cryoprotectant 2 was significantly suppressed compared to the control cells; it was also lower than the potentials of the cells from other experimental groups, but the differences were not significant (Figure 4, left side). Clonogenic potential of the passage 5 cells was similar in all the experimental groups (including cryo 2) and did not differ from that of the non-frozen cells (Figure 4, right side).

### Post-Thaw Cell Viability

As shown in Table 3, average survival of the frozen-thawed cells from the four experimental groups ranged from 77 to 92% and the differences in cell viability between the groups were not statistically significant.

**TABLE 3:** Viability of the frozen-thawed (Cryo 1 through Cryo 4) cells after their incubation for 30 min at 37°C. Mean values ± SEM are shown. The viability was performed from 5 samples.

Cryoprotectant	Viability
Cryo1	90 ± 4.1
Cryo2	92.3 ± 7
Cryo3	83 ± 11.6
Cryo4	77 ± 7.1

### Cytoskeleton Structure

**FIGURE 5:**
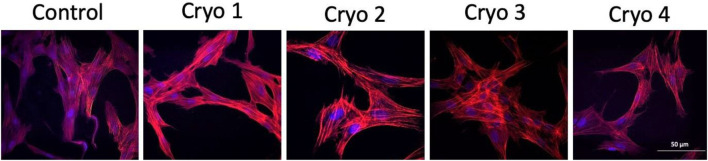
Representative images of the non-frozen (Control) and frozen/thawed (Cryo 1 through Cryo 4) AT-MSCs cells stained for the detection of actin filaments using TRITC-conjugated Phalloidin (red color) and the nucleus of the cells revealed with DAPI (blue color).

Analysis of the cytoskeleton was done because this structure helps cells maintain their shape and internal organization. By analyzing the cytoskeleton, we wanted to check whether the composition of the cryoprotective fluid may have any impact on its structure. As indicated by the actin staining test there is no such relationship. No evident differences in the microfilament structure were detected between the non-frozen (control) and frozen/thawed AT-MSCs (Figure 5).

### Differentiation Potential

**FIGURE 6:**
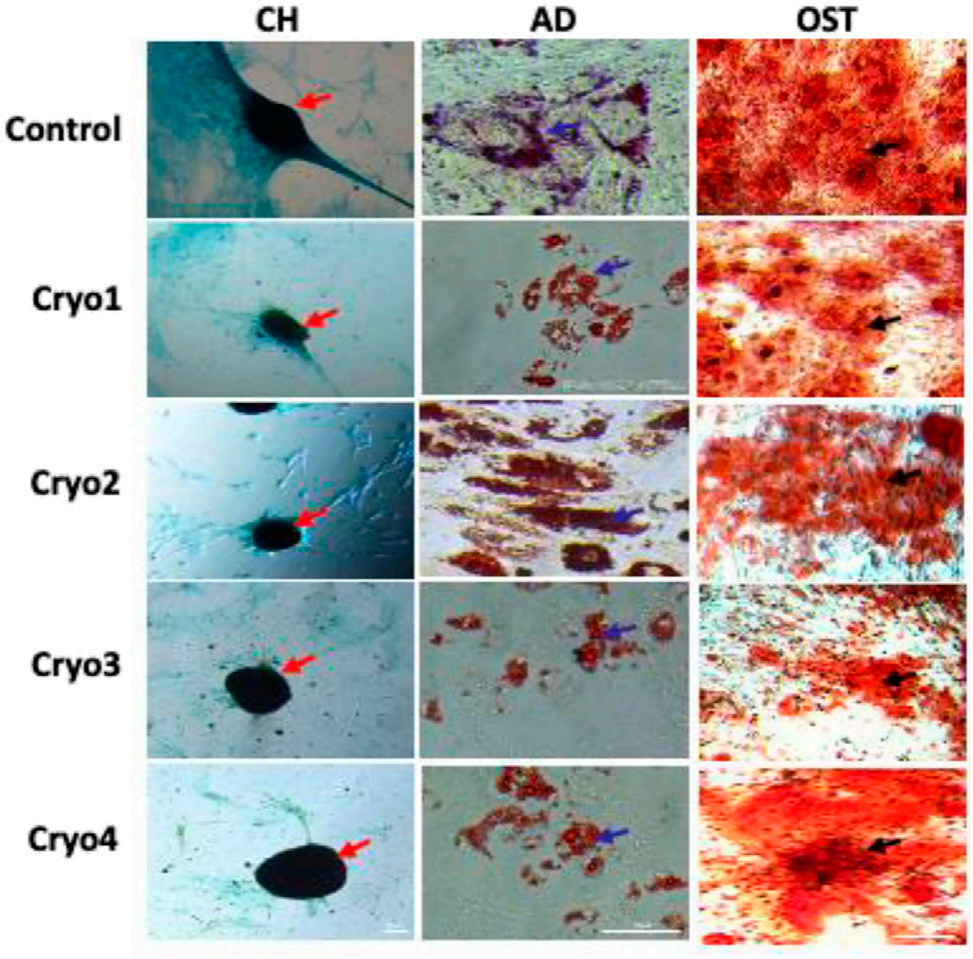
Representative images showing the potential to differentiate into chondrocytes (CH), adipocytes (AD), and osteoblasts (OST) of the non-frozen (Control) and cryoprocessed AT-MSCs. Chondrogenic differentiation was confirmed by Alcian blue staining, blue color indicates glycosaminoglycans in cartilage (red arrows); adipogenic differentiation was evaluated by Oil Red, the differentiated culture demonstrates lipid vacuoles, which typical for mature adipocytes (blue arrows); osteogenic differentiation was assessed by Alizarin Red staining of deposition of matrix calcification (black arrows).

As shown in Figure 6, none of the four cryoprotectants used affected differentiation of the AT-MSCs along the chondrogenic, adipogenic and osteogenic lineages which was similarly expressed in all the three cases (Figure 6).

### Gene Expression

**FIGURE 7:**
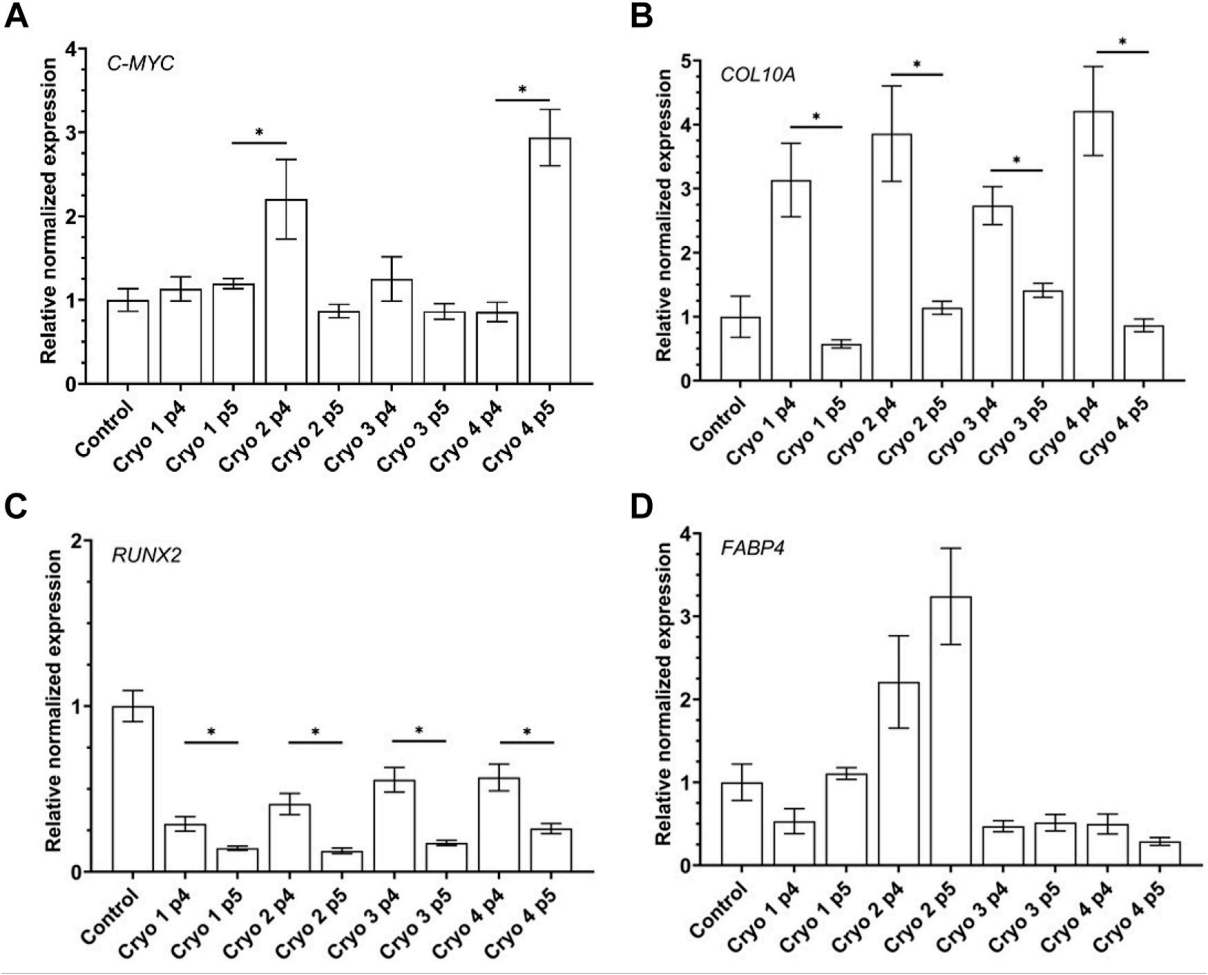
Gene expression of pluripotency related gene C-MYC **(A)** and differentiation-related genes: chondrogenic COL10A1 **(B)**, osteogenic RUNX2 **(C)**, adipogenic FABP4 **(D)** of the non-frozen (Control) and frozen/thawed AT-MSCs obtained from passages 4 and 5. Mean values ± SEM are shown; * indicates statistical significance (*p* < 0.05) of the differences between groups marked above with the horizontal solid line.

The impact of the freezing/thawing on the expression of the selected genes in the AT-MSCs is presented in Figure 7. The level of expression of *C-MYC* in the cryoprocessed cells was in most cases similar to that demonstrated by the non-processed control cells except for the cryo2 P4 and cryo4 P5 cells which exhibited the significantly increased expression of this gene (Figure 7).

Compared to the non-frozen control cells expression of *COL10A* was markedly albeit insignificantly suppressed in the cryopreserved cells from passage 5, especially in those treated with Cryo 1. In the latter cells, expression of *COL10A* was also significantly lower than in the cryopreserved cells P4 cells in which the gene was markedly but insignificantly better expressed than in the non-frozen control cells (Figure 7).

The pattern of expression of *RUNX2* was similar to that of COL10A1 as demonstrated by the significantly suppressed expression of this gene in the passage 5 cells compared to their counterparts from passage 4 from all the four experimental groups. However, compared to the control level, expression of *RUNX2* was lower in the passage 4 cells, esp. in those treated with cryo 1 and cryo2, but the differences were not statistically significant (Figure 7).

In cells treated with cryo1, cryo 3 and cryo4 expression of *FABP4* was insignificantly lower than in the non-frozen control cells. In contrast, in cells treated with cryo2 and obtained from passages 4 and 5 this expression was slightly but insignificantly higher than in the control cells (Figure 7, panel AD).

With respect to *BGLAP* no statistically significant differences in the expression of this gene were noted between the experimental groups of the cells as well as between the experimental and the control cells (data non shown).

With respect to the pluripotency-associated gene *SOX-2*, the adipogenesis-associated gene *ADIPOQ*, and the chondrogenesis-associated gene *COL2A* no expression thereof was detected in the investigated cells from both the control and the experimental groups (data not shown).

### Multiplex

As shown in Table 1, there was a noticeable variation in the secretion of IL-6, MCP-1, TGF-β1, fractalkine, G-CSF, IP-10, MIP-1α, MIP-1β, IL-10 and IFNα by the cryopreserved AT-MDCs obtained from passage 4, but generally the levels produced by these cells and that secreted by the non-frozen, control cells were not statistically significant. For example, average concentrations of IL-6 in the culture media of the cryopreserved cells ranged from 247 to 1781 pg/ml depending on the cryoprotectant used as compared to 625 pg/ml detected in the medium of the control cells. In contrast, compared to the control values, significantly lower levels of TGF-β2 and FLT-3L were detected in the media of the passage 4 AT-MDCs treated with either of the four cryoprotectants.

In the case of passage 5 cells no statistically significant differences were detected between the amounts of the tested cytokines and growth factors produced by the cryopreserved and the non-frozen, control AT-MDCs. For example, secretion of IL-6 by the cryoprocessed cells averaged from 306 to 356 pg/ml whereas in the medium of the control cells the average concentration of this cytokine equaled to 345 pg/ml. Likewise, no statistical differences could be detected between the levels of other cytokines/factors secreted by the cryoprocessed and the control cells obtained from passage 5, including TGF-β2 and Flt-3L the production of which was significantly suppressed in the frozen cells from passage 4 (Table 4).

**TABLE 4:** Cytokines secreted by the non-frozen (Control) and cryopreserved (Cryo1, Cryo2, Cryo3, Cryo4) AT-MSCs obtained from passages 4 (P4) and 5 (P5). Cytokine levels detected in the medium from the non-frozen cells served as the Control values. Mean values ± SEM are shown.

Protein/pg/ml	Control	Cryo1	Cryo2	Cryo3	Cryo4
	AT-MSCs P4				
IL-6	625.12 ± 229.06	703.28 ± 237.24	1781.56 ± 2028.14	247.91 ± 100.3	337.1 ± 170.6
IL-8	371.64 ± 68.35	265.11 ± 74.49	237.24 ± 85.92	198.65 ± 86.43	80.66 ± 38.14*
MCP-1	2,666.47 ± 1,227.97	2,102.08 ± 648.99	4,003.92 ± 2,575.54	1,410.89 ± 597.92	1,460.74 ± 521.18
TGF-β1	851.71 ± 90.08	596.94 ± 37.12	862.97 ± 263.54	755.38 ± 141.27	659.64 ± 78.28
TGF-β2	221.66 ± 16.99	46.53 ± 11.20*	59.71 ± 22.22*	47.44 ± 13.31*	34.68 ± 7.30*
PDGF AA	139.31 ± 69.10	168.36 ± 13.96	7.71 ± 0.26*	110.87 ± 10.22	92.65 ± 5.08
Fractalkine	33.64 ± 15.61	48.84 ± 30.48	22.33 ± 4.99	40.01 ± 31.33	36.77 ± 17.21
G-CSF	55.15 ± 31.17	33.01 ± 17.92	17.25 ± 8.57	27.20 ± 17.30	19.38 ± 13.35
IL-1β	1.45 ± 1.27	1.05 ± 0.34	9.30 ± 0.92*	0.80 ± 0.69	0.36 ± 0.10
IL-4	11.53 ± 4.16	6.55 ± 2.35	9.75 ± 2.53	4.35 ± 1.84	5.14 ± 2.11
IP-10	24.46 ± 14.77	6.04 ± 2.12	32.33 ± 3.44	4.71 ± 1.50	3.24 ± 1.31
MIP-1α	2.65 ± 1.01	1.52 ± 0.87	3.47 ± 1.60	1.83 ± 0.69	1.36 ± 0.77
MIP-1β	4.61 ± 2.27	1.95 ± 1.85	0.61 ± 0.36	0.35 ± 0.15	0.61 ± 0.22
Flt-3L	14.34 ± 2.32	5.36 ± 2.12*	5.07 ± 2.34*	3.90 ± 1.60*	3.92 ± 1.01*
IL-13	5.96 ± 1.73	5.40 ± 0.66	18.52 ± 1.86*	6.24 ± 1.81	5.19 ± 0.34
IL-10	2.13 ± 1.21	1.58 ± 0.37	2.36 ± 1.96	0.71 ± 0.47	0.66 ± 0.27
TNFα	6.10 ± 2.28	1.60 ± 1.05	0.90 ± 0.57	0.60 ± 0.25	0.64 ± 0.31
IFNα	2.13 ± 1.21	1.58 ± 0.37	2.36 ± 1.96	0.71 ± 0.47	0.66 ± 0.27
	AT-MSCs P5				
IL-6	345.60 ± 190.75	356.69 ± 124.05	336.70 ± 68.42	306.10 ± 73.96	356.36 ± 62.05
IL-8	44.50 ± 5.00	59.84 ± 5.45	39.12 ± 15.60	40.45 ± 17.93	56.75 ± 24.19
MCP-1	546.02 ± 203.06	780.73 ± 167.34	641.12 ± 40.04	732.53 ± 116.44	1,025.61 ± 121.07
TGF-β1	549.45 ± 156.81	633.04 ± 78.26	383.50 ± 40.74	382.94 ± 91.64	459.39 ± 90.48
TGF-β2	88.65 ± 5.20	83.56 ± 32.16	60.49 ± 19.69	60.22 ± 15.43	70.61 ± 31.27
PDGF AA	156.75 ± 51.65	111.44 ± 50.78	107.66 ± 31.16	89.54 ± 36.34	116.66 ± 48.64
Fractalkine	28.64 ± 12.37	51.25 ± 33.99	23.04 ± 18.77	12.33 ± 3.68	30.68 ± 24.66
G-CSF	31.53 ± 5.00	36.97 ± 7.00	38.71 ± 2.26	40.74 ± 1.61	34.55 ± 6.79
IL-1β	1.20 ± 0.69	0.76 ± 0.52	0.65 ± 0.30	1.32 ± 0.76	0.87 ± 0.15
IL-4	4.00 ± 1.41	5.60 ± 0.00	4.44 ± 1.63	22.72 ± 4.72*	3.56 ± 1.00
IP-10	3.44 ± 1.31	8.20 ± 3.24	3.30 ± 0.26	5.82 ± 3.33	3.15 ± 1.12
MIP-1α	1.45 ± 0.78	1.53 ± 1.00	1.36 ± 0.77	1.79 ± 1.23	1.38 ± 0.81
MIP-1β	1.10 ± 0.34	0.65 ± 0.24	0.78 ± 0.04	0.56 ± 0.25	0.75 ± 0.04
Flt-3L	6.76 ± 3.96	4.20 ± 1.94	4.27 ± 2.56	7.08 ± 4.95	4.74 ± 2.75
IL-13	3.91 ± 1.03	4.89 ± 1.22	3.88 ± 0.75	9.92 ± 0.89*	4.89 ± 1.22
IL-10	1.20 ± 0.03	1.24 ± 0.00	1.40 ± 0.21	2.63 ± 0.63	1.48 ± 0.24
TNFα	1.45 ± 1.43	0.32 ± 0.12	0.56 ± 0.41	1.49 ± 1.23	1.03 ± 0.72
IFNα	1.20 ± 0.03	1.23 ± 0.00	1.40 ± 0.21	2.62 ± 0.63	1.48 ± 0.24

*indicates statistically significant (*p* < 0.05) difference from the control value.

## Discussion

Over the recent years, mesenchymal stromal cells, including the adipose-derived MSCs, have secured a wide spectrum of clinical applications (Wang et al., 2012; Sharma et al., 2014). However, before they can be used in therapy, MSCs obtained from a certain tissue must be expanded *in vitro* to the appropriate number and then stored until their quality studies are completed and the cells are released as a medical product. In order for the cells to be stored safely and for a long time, they must be subjected to deep-freezing. That is why cryopreservation, as one of the crucial steps in the preparation of MSCs, must be optimized and applied as a safe and thoroughly mustered technique. As was previously indicated, neither the recovery rate, viability nor phenotype of MSCs were seriously affected by thawing (Luetzkendorf et al., 2015; Pollock et al., 2015; Oja et al., 2019), the observation confirmed by us in the present study. It is also important, however, to assess physiological stability of the frozen-thawed cells and to estimate the impact of various freezing conditions on the cells’ properties. Hence, in the present investigation four different DMSO-based cryoprotectants were employed to check their effect on the physiological properties of the cells after thawing and to select the optimal freezing formula.

As indicated by the present results, none of the cryoprotectants markedly affected the shape, adhesion to plastic or the cytoskeleton structure of the tested cells. Likewise, no statistically significant differences between the cells from the experimental and control groups were detected in the rate of proliferation and the colony-forming capacity of these cells, as indicated by the results of the PDT and CFU assays, respectively.

As demonstrated by Shaik and co-workers cryopreservation of the adipose tissue-derived stem cells can be successfully performed for as long as a decade. These authors found that the viability of MSCs thawed after the 10 years storage remained at about 78% which was not significantly less compared to the cells kept frozen for a shorter time (Shaik et al., 2018). In accord with this the viability of our AT-MSCs ranged from 78 to 90% although we stored the cells markedly shorter. On the other hand, it was demonstrated that long-term storage of hematopoietic cells at −150°C did not affect the cells’ viability proliferative potential (Broxmeyer et al., 2003; Broxmeyer et al., 2011; Kim et al., 2021). Some authors (Oja et al., 2019) demonstrated a better than our viability of the frozen-thawed cells, but only in cells obtained from the first passages. Overall, our present results confirm those of other authors’ indicating that freezing of MSCs does not markedly disturb the viability of the frozen-thawed cells (Luetzkendorf et al., 2015; Shaik et al., 2018; Oja et al., 2019).

In our present study, the phenotype of the frozen-thawed AT-MSCs did not differ from that of the non-frozen, control cells, irrespectively of the cryoprotectant or cell passage used. Variation in the homogeneity of our cell populations with respect to the expression of hematopoietic markers did not exceed 1% and was similar to or better than that reported by others (Mitchell et al., 2006; Baer et al., 2013). Moreover, our present findings, in accord with those of others’ (Luetzkendorf et al., 2015; Oja et al., 2019), indicate that freezing of MSCs does not affect the phenotype of these cells. In fact, although expression of CD105 which was low just after thawing returned to normal during passages 4 and 5.

To confirm the multilineage potential of the cells under study we checked the expression of genes whose function is associated with osteogenesis, chondrogenesis and adipogenesis as well as those associated with pluripotency. We found that in all the cryopreserved (experimental) groups of cells expression of the osteogenesis-associated gene *RUNX2* was substantively muted when the cells were obtained from the passage 5 compared to those from passage 4. In turn, the latter cells expressed less *RUNX2* than did the non-frozen control cells, but the difference was not statistically significant. These findings are in agreement with those of James and co-workers (James et al., 2011). With respect to adipogenesis we found that expression of the *FABP4* gene was significantly down-regulated in cells from all the experimental groups except for those treated with cryoprotectant 2. In the latter case expression of *FABP4* was similarly elevated in cells obtained from passages 4 and 5. These results vary from those of Zanata and co-workers who did not detect any decrease in the adipogenic potential of the cryopreserved adipose tissue cells (Zanata et al., 2018). In the present study, no significant differences were noted in the chondrogenic potential (as estimated by the expression of the *COL10A1* gene) of cells treated with the four cryoprotectants, although there were significant differences between the passage 4 and passage 5 cells. In contrast, James et al. reported that the chondrogenic potential of frozen AT-MSCs was lower than that of “fresh” MSCs (James et al., 2011). With respect to the expression of *C-MYC* we have observed its inexplicable up-regulation in the cryo2 P4 and the cryo 4 P5 cells. Unfortunately, in our hands the expression of the genes’ tested did not coincide with the cells’ differentiation capacities *in vitro*; there were no differences between the cells from the experimental groups obtained from the 4th and the 5th passage. Moreover, visualization of the differentiated cells did not reveal any significant differences between the experimental and control groups. At present, we have no plausible explanation for the observed discrepancies between the expression of the selected genes and the capacities of the tested cells to undergo differentiation into adipocytes, chondrocytes, or osteocytes.

In the present investigation, the secretory properties of AT-MSCs appeared to be similar to the those reported by other authors (Kilroy et al., 2007; Krumboeck et al., 2013). We have demonstrated that although the capacity to produce cytokines and growth factors might be compromised in freshly thawed cells, eventually the whole process of freezing, storage and thawing of AT-MSC did not significantly impair the secretory function of these cells. This secretory instability occurring in some experimental groups and in relation to some factors seems to be quite random and it is difficult to see any regularity in this phenomenon. Indeed, the temporarily unstable secretion of almost all of the tested factors by cells obtained from passage 4 was fully restored during the 5th passage, regardless of the cryoprotectant used. Similar results were reported by other authors who showed that impairment of the secretory factors-mediated immunomodulatory properties of MSCs, which occurred immediately after thawing (François et al., 2012; Moll et al., 2014), disappeared quickly in culture (François et al., 2012). According to these authors the early post-thaw drop in the cells’ secretory function is associated with the cryopreservation-induced heat-shock stress. Our present results support this notion, although the secretory function of the cells investigated by us returned to normal after a slightly longer period of time. Nevertheless, the heat-shock stress that occurs in the cell after freezing/thawing procedure seems to be a factor which, although undetectable in classical cellular tests, is clearly visible in the physiology of cells and may quite significantly affect their properties immediately after thawing.

Unfortunately, in the majority of clinical applications of AT-MSC, these cells are administered directly after thawing. Our observations show that this procedure physiologically destabilizes the cells, even if the routine qualitative tests (viability, recovery, phenotype) do not reveal this. It cannot be ruled out that the transient physiological instability may affect the therapeutic effectiveness of the applied cells, especially in terms of their secretory capacities. A simple and low-cost solution to this problem may be stabilitization of the cells in a short culture what, as clearly demonstrated in our investigation, restores their secretion capacity and, to a lesser extent, expression of the selected genes immediately after thawing and later in the culture. The composition of the applied cryoprotective fluid did not seem to affect this phenomenon in any way. All these observation allows us to draw two basic conclusions:

- judging by the dynamics of changes of the secretory function of the thawed AT-MSCs we suggest that before their clinical application the cells should be (if possible) cultured for at least one passage to recover their physiological stability and thus assure their optimal therapeutic potential;
- DMSO-based cryoprotectants are safe and efficient agents for freezing of AT-MSCs and as such can be used in many variants.

## Data Availability

The original contributions presented in the study are included in the article/Supplementary Material, further inquiries can be directed to the corresponding author.

## Abbreviations

AT-MSCs, adipose tissue-derived mesenchymal stromal cells; ATP, adenosine triphosphate; BM-MSCs, bone marrow-derived mesenchymal stromal cells; CFU-F, colony-forming unit fibroblast; Cryo, cryoprotectant; DAPI, 4′,6-diamidino-2-phenylindole; DMSO, dimethyl sulfoxide; FITC, fluorescein isothiocyanate; MSCs, mesenchymal stromal cells; P, passage; PDT, population doubling time; PE, phycoerythrin; SVF, stromal vascular fraction; TRITC, tetramethylrhodamine.
